# Risk-Taking Behavior in a Computerized Driving Task: Brain Activation Correlates of Decision-Making, Outcome, and Peer Influence in Male Adolescents

**DOI:** 10.1371/journal.pone.0129516

**Published:** 2015-06-08

**Authors:** Victor Vorobyev, Myoung Soo Kwon, Dagfinn Moe, Riitta Parkkola, Heikki Hämäläinen

**Affiliations:** 1 Centre for Cognitive Neuroscience, Turku Brain and Mind Center, Department of Psychology, University of Turku, Turku, Finland; 2 Department of Transport Research, SINTEF Technology and Society, Trondheim, Norway; 3 Department of Radiology, University Hospital of Tampere, Tampere, Finland; University of Pennsylvania, UNITED STATES

## Abstract

Increased propensity for risky behavior in adolescents, particularly in peer groups, is thought to reflect maturational imbalance between reward processing and cognitive control systems that affect decision-making. We used functional magnetic resonance imaging (fMRI) to investigate brain functional correlates of risk-taking behavior and effects of peer influence in 18–19-year-old male adolescents. The subjects were divided into low and high risk-taking groups using either personality tests or risk-taking rates in a simulated driving task. The fMRI data were analyzed for decision-making (whether to take a risk at intersections) and outcome (pass or crash) phases, and for the influence of peer competition. Personality test-based groups showed no difference in the amount of risk-taking (similarly increased during peer competition) and brain activation. When groups were defined by actual task performance, risk-taking activated two areas in the left medial prefrontal cortex (PFC) significantly more in low than in high risk-takers. In the entire sample, risky decision-specific activation was found in the anterior and dorsal cingulate, superior parietal cortex, basal ganglia (including the nucleus accumbens), midbrain, thalamus, and hypothalamus. Peer competition increased outcome-related activation in the right caudate head and cerebellar vermis in the entire sample. Our results suggest that the activation of the medial (rather than lateral) PFC and striatum is most specific to risk-taking behavior of male adolescents in a simulated driving situation, and reflect a stronger conflict and thus increased cognitive effort to take risks in low risk-takers, and reward anticipation for risky decisions, respectively. The activation of the caudate nucleus, particularly for the positive outcome (pass) during peer competition, further suggests enhanced reward processing of risk-taking under peer influence.

## Introduction

Adolescence, a critical transition period of physical and psychological development between childhood and adulthood, is characterized by novelty-seeking and risk-taking behavior [1]. Adolescents also have a stronger motivation for peer acceptance than children and adults [2, 3]. Thus, adolescent risk-taking is much more likely to occur in the presence of peers, as evidenced in reckless driving [4], substance abuse [5], and crime [6]. Adolescents also took more risks in peer groups in experimental situations [7, 8].

Adolescents’ relatively greater risk-taking propensity is thought to reflect maturational imbalance between two competing brain systems that affect decision-making: reward processing and cognitive control systems [1, 9–16]. The reward processing system involving the ventral striatum (including the nucleus accumbens, NAcc) and orbitofrontal cortex shows a dramatic change in early adolescence, while the cognitive control system involving the lateral prefrontal (PFC) and dorsal anterior cingulate cortices (ACC) undergoes relatively gradual maturation. Thus, increased sensitivity to rewards, paired with immature cognitive control ability to down-regulate the reward system, may bias adolescents’ decisions toward greater risk-taking. Given the elevated reward value of peer interactions in adolescence [2, 3], the presence of peers may further sensitize the reward system to potential rewards of risky behavior.

In a recent study [17] using functional magnetic resonance imaging (fMRI), the influence of peers on adolescents’ decisions was reflected in the increased activation of reward-related brain regions, including the ventral striatum and orbitofrontal cortex, and the activity predicted subsequent risk-taking in a simulated driving task. Brain areas associated with cognitive control were less strongly recruited by adolescents than adults, and the activity did not vary with peer influence. This suggests that the presence of peers increases adolescent risk-taking by increasing sensitivity to potential rewards of risky decisions.

Risk-taking behavior during adolescence can be predicted by relevant personality traits. Recent studies found significant correlations between questionnaire measures of personality traits (e.g., sensation-seeking, impulsivity) and behavioral measures of cognitive tasks [18], or simulated [19] or self-reported [20] risky driving behavior in adolescents. However, few studies considered the traits associated with susceptibility to peer influence, and risk-taking behavior in the face of peer pressure.

It is known that adolescents are more likely to engage in risky driving behavior than adults, particularly in the presence of peers, and relevant age-related differences in brain activation have been found [17], while brain mechanisms underlying variation in risk-taking behavior among adolescents of the same age group, assuming minimal differences in brain maturation, remain to be studied. Therefore, we recruited adolescents of a narrow age range (18–19 years old) consisting of low and high risk-taking groups assessed by personality tests, and compared the behavior and brain activation of the two groups as they performed a modified version of the computerized driving task used in [17]. Specifically, we examined differences in brain activation when the adolescents made risky (Go) or safe (Stop) decisions at a series of intersections with traffic lights, and faced the outcomes (Pass or Crash) of the risky decision. We made comparisons in the same way between groups formed by actual risk-taking behavior during task performance. Finally, we tested effects of peer influence on decision- and outcome-related activation by including a peer competition situation.

## Materials and Methods

### Subjects

Personality tests were administered to 215 students at a local vocational school. In 187 right-handed male respondents (excluding females and/or left-handers), 43 high and 46 low risk-takers (with low and high resistance to peer influence, respectively) were selected, and 17 from each group agreed to participate as subjects of the study. The subjects were 18–19 years old with no history of neurological or psychiatric problems, and no sign of pathology in visual inspection of their anatomical head scans.

The study was approved by the Ethics Committee of the Hospital District of Southwest Finland, and conducted according to the Declaration of Helsinki. The subjects gave written informed consent and were paid (150 euro) for their participation.

### Personality tests

The tests included Zuckerman-Kuhlman-Aluja Personality Questionnaire (ZKA-PQ) [21] and The Resistance to Peer Influence Scale (RPIS) [22]. In the ZKA-PQ, the items that represent sensation-seeking (4 facets: thrill- and adventure-seeking, experience-seeking, disinhibition, boredom susceptibility/impulsivity) and neuroticism (2 facets: dependency, low self-esteem) factors were used to assess risk-taking propensity and susceptibility to peer influence, respectively. The ZKA-PQ included 60 items, 10 items for each facet, while the RPIS included all 10 items. The tests were translated from English to Finnish by a professional translator, and administered online using Webropol 2.0 (Helsinki, Finland).

Through the questionnaire, we also obtained driving-related background information on whether they drive a vehicle (moped, motorcycle, car, truck, etc.), had an accident, play driving games, and which line of study they pursue.

### Driving task

**Fig 1:**
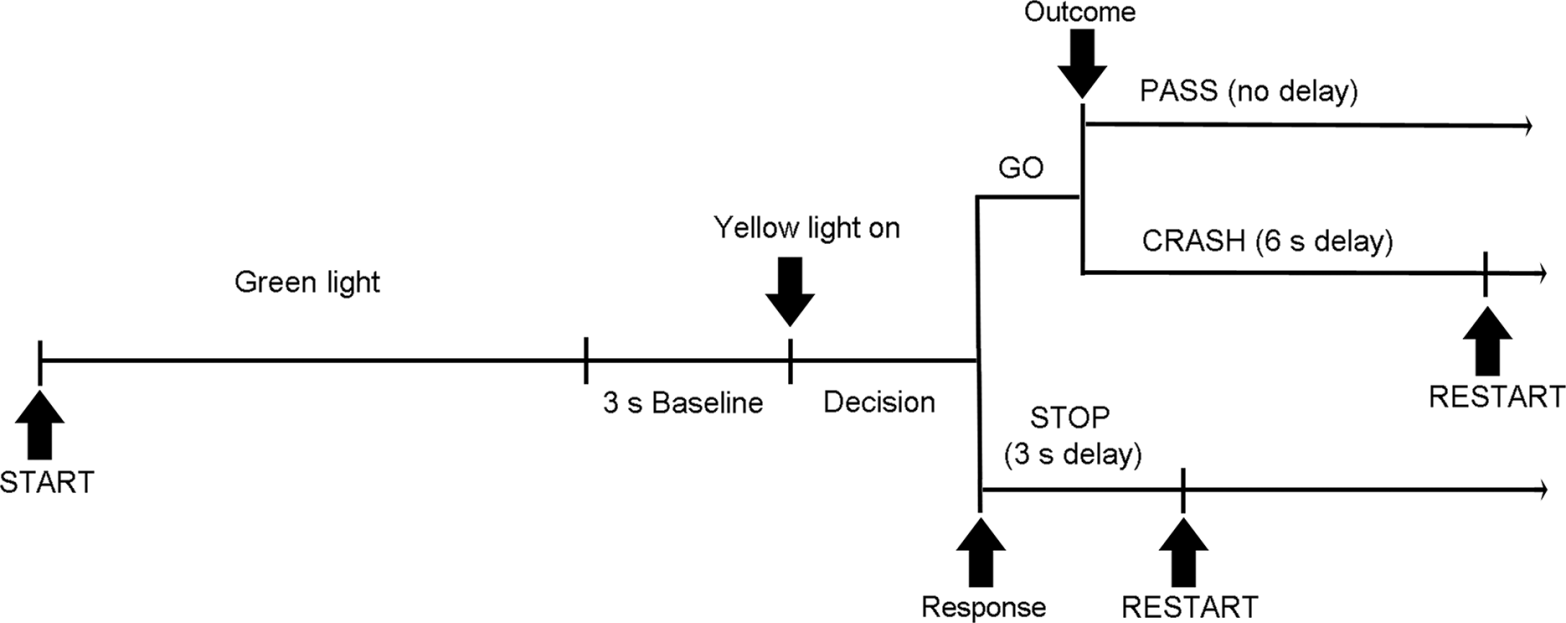
Timeline (from left to right) of events in a single round (trial) of the Stoplight game.

We used a modified version of the Stoplight Game program [17], in which a car from a driver’s point of view moved along a straight track through 20 intersections with traffic lights (Fig 1 and S1 Fig), with the goal of reaching the end of the track as quickly as possible within 5 min. As the car approached the intersection, the traffic light turned yellow and the subject made a decision whether to stop and wait for a red light to turn green (with a short 3-s delay), or to go through the intersection without braking (for no delay) and take the risk of a car crash (with a longer 6-s delay). The decision was made after the light turned yellow by pressing Stop or Go button placed under the index and middle finger of the right hand, respectively, being on the same side of the brake and accelerator pedals of a car.

The traffic light at the first intersection remained green, and the other 19 trials were used for data analysis. The distance between intersections (i.e., inter-trial interval) varied at 11–13 s, and the traffic light turned yellow 1.5–3.0 s before entering an intersection, and turned red 0.5 s before the intersection. Another car, invisible in advance, crossed the track either immediately or 2.0 s after the subject’s car arrived at the intersection, but the probability of the inevitable crash for Go decisions was constant across sessions (7 trials without delay), and associated with 1.5–2.5, but not 3.0-s duration of yellow light. We prepared six variants of the game parameter sets, each with its own predefined arrangement of traffic light timing and intersection positions associated with a crash. Two of them were for practice sessions and the other four were presented in actual experiments in a counterbalanced order across subjects.

In practice, a crash could occur if the Stop button was pressed too late and the car stops in the middle of the intersection, even when another car crossed the intersection in 2 s. If the button was pressed more than once, the game program lets the car cross the intersection without stopping, leading to an unwanted crash. Thus, the subject was instructed to press the button at the right moment and to avoid repetitive button press, and very few such mistakes did not affect the results of the experiment.

Behavioral data (Stop button press) and other task events were registered by the Stoplight Game program, which, in turn, was controlled by another computer running the Presentation software (Neurobehavioral Systems, Albany, CA) to synchronize the task events and fMRI data acquisition and to register Go responses that was not supported by the Stoplight Game program. The game program also included crashing and braking sounds, and a simple music that gradually increased in loudness, but the subject could hear them only during practice sessions outside the MRI scanner.

### Procedure

The experiment was conducted at the Department of Radiology of the Turku University Hospital (TYKS), Turku, Finland. The subject was given instructions and learned the task by performing a short 10-trial guided game sitting in a chair outside the MRI room. After two full-length practice sessions, the subject was placed in an MRI scanner and completed four sessions of the task under two different social conditions, the first two sessions in the non-competition condition and the other two sessions in the competition condition. In the non-competition condition, the subject was instructed only to reach the end of the track as quickly as possible, the same as in the practice sessions, while in the competition condition, the subject was instructed that the performance results of the current session would be presented at school and compared among their peers to reveal the fastest record. The change of social condition was a surprise manipulation, and the last two sessions were presumably performed under social pressure of a peer competition situation.

It took less than 1 h to complete six sessions of the task (including two practice sessions), and the whole experiment including instructions, positioning in the scanner, and structural MRI scans before and after task performance took about 2 h. After the experiment, we asked the subject about the timing of decision-making, whether the competition situation had effects on decisions, and whether the game was boring, neutral, or catching.

#### MRI data acquisition

Subjects were scanned with a 3-Tesla Siemens (Erlangen, Germany) Magnetom Verio MRI scanner equipped with a head coil. Each of four fMRI sessions included 195 whole brain volume acquisitions of blood-oxygenation-level dependent (BOLD) signal using a single-shot sequence for T2*-weighted echo planar imaging with 2.0 s TR, 30 ms TE, 70° flip angle, 80 × 80 matrix, 33 slices, 3.45 mm slice thickness including 0.45 mm gap, and 3 × 3 mm in-plane resolution. In addition, a magnetization-prepared rapid-acquisition gradient echo (MPRAGE, 2.3 s TR, 3.4 ms TE, 256 × 200 matrix) scan was collected to obtain a high-resolution (1 mm^**3**^ voxel) 3D structural T1-weighted image of the whole head for medical inspection and data analysis.

The game view was projected to a semitransparent screen that could be seen through a mirror mounted on the head coil. The start of each game session was synchronized with the start of fMRI data acquisition by a computer running the Presentation software, which triggered the other computer running the Stoplight game program.

### Data analysis

#### Questionnaire data

Both ZKA-PQ and RPIS used a four-point scale scored from one to four, including reverse-scored items, and the sum of scores was used for participant selection and data analysis. The relationship between sensation-seeking scores and risk-taking behavior (Go response rates), and between neuroticism or RPIS scores and peer influence (differences in Go response rates between social conditions) was assessed with a bivariate correlation (one-tailed).

Because the sum of scores could not predict risk-taking behavior during task performance (see Results), the scores of individual items were also examined whether any of them better discriminate performance-based low and high risk-takers using an item discrimination index and an independent-samples t-test (one-tailed). The formula for the discrimination index was D = U/n_u_−L/n_l_, where n_u_ is the number of people in the upper group, U is the number of people in the upper group who scored high on the item, n_l_ is the number of people in the lower group, and L is the number of people in the lower group who scored high on the item [23]. Specifically, we measured whether the upper group who took more risks (i.e., performance-based high risk-takers, n_u_ = n_l_ = 17) scored high (3 or 4) on the ZKA-PQ item, and whether the upper group who took more risks in the competition condition (n_u_ = 20, n_l_ = 14) scored low (1 or 2) on the RPIS item. The discrimination index range between -1 and 1, and the D value of 3.0 or above was considered good at discrimination [23].

#### Behavioral data

Subjects were initially divided into low and high risk-taking groups based on questionnaire data, but there was a discrepancy in risk-taking behavior between questionnaire results and actual task performance (see Results). Thus, the number of risky decisions (Go responses) actually made during task performance was used as an alternative criterion for grouping.

The number of Go and Stop responses and their response time (from yellow light onset to button press) were obtained from four MRI sessions with 19 trials each. The Go response (or risk-taking) rate was compared using a three-way repeated-measures analysis of variance (ANOVA) with Peer (competition, non-competition), Session (first and second within each condition), and between-subject Group (low and high risk-taking) factors. Both Go and Stop response (or decision-making) time was compared using a four-way ANOVA with Decision (Go, Stop), Peer, Session, and Group factors. The significance level was set at 0.05.

The number of Pass and Crash following Go decisions, and the number of Go and Stop decisions following either outcome were also obtained from the same data and compared with a five-way ANOVA with Outcome (Pass, Crash), Decision (Go and Stop following either outcome), Peer, Session, and Group factors.

The ANOVA of questionnaire- and performance-based groups only showed difference for the main effect of Group. Thus, we mainly reported performance-based ANOVA, while briefly mentioning the main effect of Group for questionnaire-based ANOVA.

#### MRI data

Seven subjects were excluded from fMRI data analysis because they violated instructions or made only (or almost only) Stop responses (n = 3), reported loss of interest in playing the game due to annoying MRI noise (n = 1), or due to incomplete data or imaging artefacts (n = 3). Thus, the data analysis was performed in 27 subjects, and the ratio of questionnaire- and performance-based low and high risk-taking groups was 14/13 and 11/16, respectively.

The MRI data were processed with the Statistical Parametric Mapping (SPM8, Wellcome Department of Cognitive. Neurology, London, UK; http://www.fil.ion.ucl.ac.uk/spm) implemented in Matlab (MathWorks Inc., Natik MA, USA). First, between-scan head motions were corrected by realigning all functional scans to the first one in the series using a six-parameter rigid body transformation. The mean realigned image and transformation parameters were recorded. Then, the high-resolution structural image was coregistered in space to the mean realigned image and segmented into tissue classes. The resulting image of gray matter was warped to the SPM8 gray matter MRI template corresponding to the Montreal Neurological Institute (MNI) coordinate space. The transformation parameters recorded at this step were then applied to the functional images, which were also resliced to 3 mm cubic voxel. Finally, the normalized functional images were smoothed with an 8 mm (full width at half maximum) isotropic Gaussian kernel.

Statistical analysis was done as a two-level procedure by extracting subject-specific contrasts of interest at the first level and entering them into the second-level group analyses. At the first level, BOLD signal changes during task performance were modeled as a sequence of conditions/events convolved with a hemodynamic response function using a General Linear Model (GLM) implemented in the SPM8. The decisions (Go or Stop) were modeled as variable length epochs from yellow light onset to button press, corresponding to response time [24]. The outcomes (Pass or Crash) following Go decision were modeled with a stick function as events. The 3-s period preceding the onset of yellow light served as a baseline.

The model also included six realignment (rigid body transformation) parameters to regress out residual head motion-related signal changes. A high-pass filter with a cut-off period of 128 s was applied to account for a low frequency scanner drift. Effects of interest, in the form of between-condition contrasts, were obtained for each subject and the contrast images were then used in the second-level between-subject analysis.

At the second level, first, differences in activation between Go and Stop decision, and Pass and Crash outcome were obtained for the entire pool of subjects in a series of t-tests treating subjects as a random factor and using a p < 0.05 voxel level significance threshold after family-wise error (FWE) correction based on the Gaussian random field theory. Then, the effects of grouping were investigated by testing the interactions between Group and Decision or Outcome factors with a within-subject GLM-based ANOVA using a flexible factorial design and contrasts with Baseline as input data. The Subject factor was also included into the model. For significant interactions (voxel-wise p < 0.05, FWE corrected), post-hoc t-tests were performed to reveal directions of local activation effects. Similarly, the interaction between Group and Peer factors was tested with a 2 × 2 ANOVA using direct contrasts between the decision types or the outcome types as input data. Since no group-specific significant interaction was found (see Results), the data were combined across groups and the test of interaction was repeated for the entire pool of subjects with two within-subject factors: Peer and Decision or Outcome.

In some cases (see Results), in order to perform additional region of interest (ROI) analyses with small volume correction, as well as to show plots of local effect size across conditions, we built 6-mm spherical regions around peaks of effects and built final ROIs by extracting the overlap between the spheres and the analysis mask. The mean activity across voxels in each ROIs was extracted with the Marsbar toolbox for SPM [25].

Brain structures corresponding to the MNI coordinates of activation peaks were defined in the stereotactic space [26] after applying the mni2tal correction algorithm [27]. Cerebellar responses were localized with the cerebellar probabilistic atlas [28].

## Results

### Questionnaire data

The initial grouping of low and high risk-takers according to their personality test results (S2 Fig) did not predict their actual risk-taking behavior in the driving task (S3 Fig). The correlation between test scores and risk-taking (Go response rates) or peer influence (differences in Go response rates between social conditions) was not significant in predicted directions (one-tailed). Most of the items could not discriminate subjects according to their risk-taking (ZKA-PQ: mean D = 0.01, range: -0.29 ~ 0.35) and peer influence (RPIS: mean D = -0.07, range: -0.31 ~ 0.24) during task performance. Only two ZKA-PQ items showed D values over 3.0: #107. I enjoy many types of loud, intense rock music (D = 0.35), and 137. I don’t like to start a project until I know exactly how to proceed (D = 0.35). The t-test revealed significant differences in three RPIS items (#1, 3, and 5; one-tailed), but only in the opposite direction: Those who scored more resistant to peer influence took more risks (Go) in the peer-competition situation.

### Behavioral data

#### Decision phase

Go response rates of individual subjects averaged over the four sessions of the task corresponded to a normal distribution (Kolmogorov-Smirnov Z = 0.480, p = 0.975; mean = 7.903, SD = 3.84), and one half of them with Go response rates exceeding a median value of 8.5 were grouped as high risk-taking, and the other half as low risk-taking. The number of Go response, and Go and Stop response time for performance-based groups and social conditions are provided in Table 1.

**Table 1:** The mean (SD) number of Go responses, Pass and Crash outcomes, and Go and Stop decisions after either outcome for performance-based groups and social conditions.

	Non-competition		Competition	
Task events	Low risk-takers	High risk-takers	Low risk-takers	High risk-takers
Go	4.1 (2.5)	10.5 (2.6)	5.4 (2.6)	11.4 (2.4)
Go RT	0.42 (0.18)	0.47 (0.11)	0.51 (0.23)	0.53 (0.16)
Stop RT	0.47 (0.10)	0.48 (0.11)	0.52 (0.14)	0.54 (0.15)
Pass	2.5 (1.3)	6.4 (1.8)	3.6 (1.8)	7.1 (1.6)
Go after Pass	0.3 (0.4)	2.4 (2.1)	0.9 (1.0)	3.4 (1.9)
Stop after Pass	2.2 (1.1)	3.8 (1.2)	2.7 (1.2)	3.5 (0.9)
Crash	1.7 (1.0)	4.1 (1.1)	2.0 (1.2)	4.4 (1.1)
Go after Crash	0.3 (0.4)	2.2 (1.1)	0.6 (0.6)	2.3 (1.5)
Stop after Crash	1.2 (0.8)	1.6 (0.6)	1.2 (0.8)	1.6 (1.0)

Go and Stop response time is also provided under the row for Go responses.

The main effect of Peer was significant for the Go response rate (F_1, 32_ = 8.580, p = 0.006): The Go response rate was higher in the competition (mean = 8.426, SE = 0.431) than the non-competition (mean = 7.324, SE = 0.415) condition. The main effect of Group was also significant for the Go response rate (F_1, 32_ = 67.257, p < 0.0005): The Go response rate was higher in the high (mean = 10.985, SE = 0.536) than the low (mean = 4.765, SE = 0.536) risk-taking group. The main effect of Group was not significant for questionnaire-based groups. The interaction between Peer and Group was not significant. The main effect of Peer was significant for response time (F_1, 28_ = 12.866, p = 0.001): Both Go and Stop response time was longer in the competition (mean = 0.529, SE = 0.027) than the non-competition (mean = 0.465, SE = 0.018) condition. The main effect of Decision and Group, and the interaction between Decision, Peer, and Group were not significant.

Group comparisons according to other criteria (whether they drive a vehicle, had an accident, play driving games, pursue a traffic-related line of study, or had any difference in decision-making between conditions) only revealed a higher Go response rate (mean = 9.617, SE = 0.928) in the group that reported as having had a difference in decision-making between the competition and non-competition conditions than in the other group (mean = 6.412, SE = 0.872; F_1, 30_ = 6.335, p = 0.017).

#### Outcome phase

The number of Pass and Crash, and Go and Stop decisions following either outcome for performance-based groups and social conditions are provided in Table 1.

The main effect of Outcome was significant (F_1, 32_ = 146.116, p < 0.0005): The number of Pass (mean = 2.382, SE = 0.123) was higher than that of Crash (mean = 1.338, SE = 0.074). The main effect of Decision (following either outcome) was significant (F_1, 32_ = 8.173, p = 0.007): The number of Stop decision (mean = 2.191, SE = 0.112) was higher than that of Go decision (mean = 1.529, SE = 0.177). The main effect of Peer was significant (F_1, 32_ = 8.784, p = 0.006): The number of either outcome was higher in the competition (mean = 2.011, SE = 0.105) than the non-competition (mean = 1.710, SE = 0.105) condition due to the higher Go response rate in the competition condition. The main effect of Group was significant (F_1, 32_ = 59.373, p < 0.0005): The number of either outcome was higher in the high (mean = 2.570, SE = 0.130) than the low (mean = 1.151, SE = 0.130) risk-taking group due to the higher Go response rate in the high risk-taking group. The main effect of Group was not significant for questionnaire-based groups.

The interaction between Outcome and Decision following either outcome was of particular interest, and significant (F_1, 32_ = 17.872, p < 0.0005): The number of Stop decision was higher than that of Go decision after Pass, while the number of either decision was small after Crash. The interaction between Outcome, Decision, and Group or Peer was not significant. The interaction was significant between Outcome and Group (F_1, 32_ = 14.674, p = 0.001), Outcome and Peer (F_1, 32_ = 6.686, p = 0.014), Decision and Group (F_1, 32_ = 7.290, p = 0.011), and Decision and Peer (F_1, 32_ = 4.838, p = 0.035): The relationship between the factors can be specified by the number of task events provided in Table 1.

### MRI data

#### Decision phase

**Fig 2:**
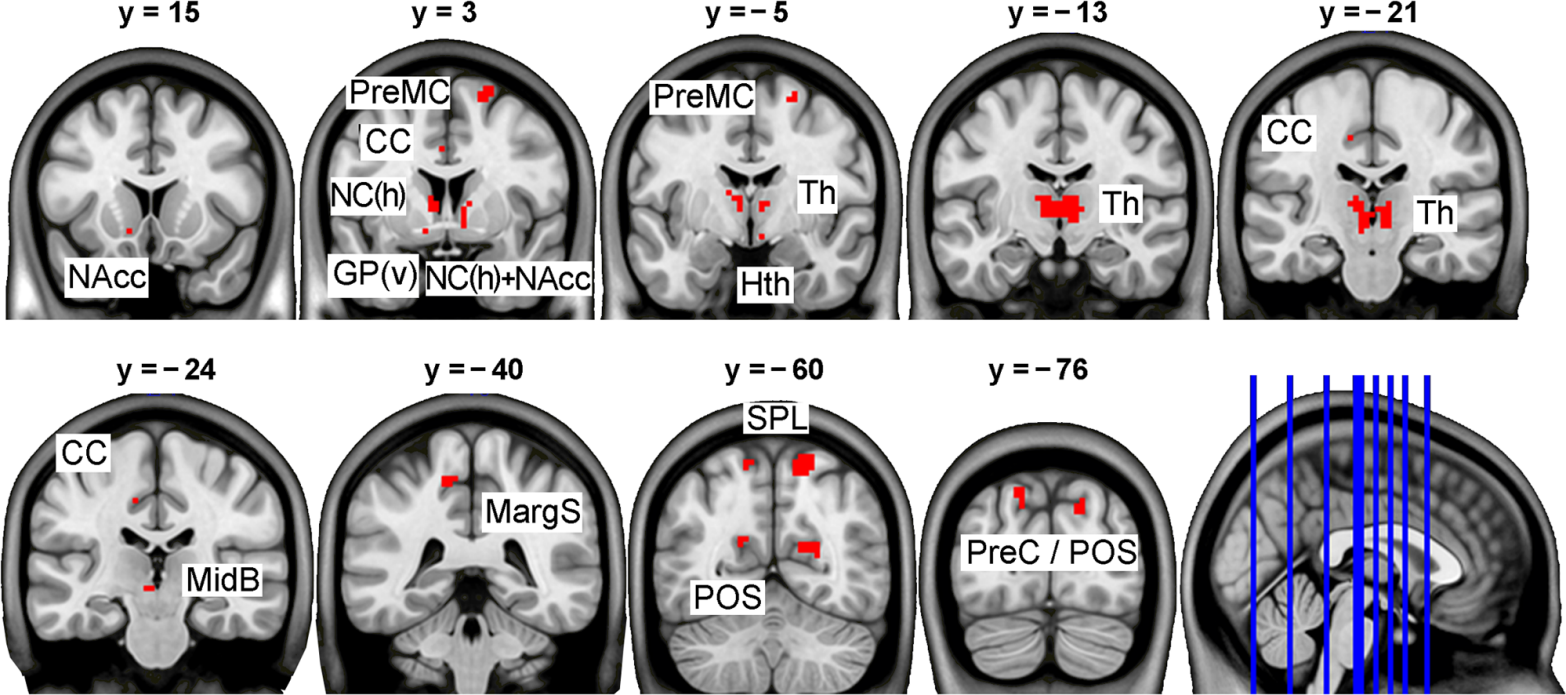
Areas of stronger activation for Go than Stop decision (p < 0.05, FWE corrected). The areas are shown overlaid onto a series of sample coronal slices of the MNI152 template specified with y coordinates above the slice images and in the lower right sagittal image. The right hemisphere corresponds to the right half of the coronal images. Statistical and location details of the activation peaks are provided in Table 2. CC: cingulate cortex; GP(v): ventral globus pallidus; Hth: hypothalamus; MargS: marginal sulcus; MidB: midbrain; NAcc: nucleus accumbens; NC(h): head of caudate nucleus; POS: parietooccipital sulcus; PreC: precuneus; PreMC: premotor cortex; SPL: superior parietal lobule; Th: thalamus.

A direct comparison between Go and Stop decisions revealed a predominance of activated areas in the Go > Stop contrast (Fig 2, Table 2), comprising the dorsal premotor, dorsal cingulate, superior parietal, and parieto-occipital cortical areas. Subcortical clusters were found in the anterior and ventral parts of the striatum (including the NAcc) bilaterally, in the left ventral pallidum, right hypothalamus, left midbrain, and in the anterior and medial thalamus in both hemispheres. The opposite Stop > Go contrast revealed only two left occipital peaks.

**Table 2:** Peak activation differences between conditions in the decision phase.

t-contrast	BA	t	p _FWE-corr._	MNI coordinates		
Brain structure				x	y	z
**Go > Stop**						
R. Sup. Frontal Gyrus	6	8.09	<0.001	21	-1	64
R. Post. Cingulate Sulcus	31	7.42	0.002	21	-58	16
L. Cingulate Sulcus	24/31	6.01	0.038	-12	-22	43
L. Ant. Cingulate Gyrus	24	6.18	0.026	-6	5	34
L. Ant. Cingulate Gyrus	24	6.09	0.032	-3	2	37
L. Marginal Sulcus	5/7	6.77	0.008	-12	-40	55
R. Sup. Parietal Lobule	7	7.63	0.001	18	-64	61
R. Sup. Parietal Lobule	7	7.23	0.003	15	-55	61
L. Sup. Parietal Lobule	7	6.27	0.022	-15	-64	64
L. Sup. Parietal Lobule	5/7	7.15	0.003	-21	-49	61
L. Precuneus	7	6.71	0.009	-15	-76	46
R. Postcentral Gyrus	5/7	6.35	0.019	15	-49	73
L. Postcentral Gyrus	5/7	6.79	0.007	-18	-49	73
R. Parieto-occipital Sulcus	7/19	7.45	0.002	18	-76	37
R. Parieto-occipital Sulcus	31	7.42	0.002	21	-58	16
L. Parieto-occipital Sulcus	31	6.09	0.032	-18	-61	22
L. Nucleus Accumbens		6.08	0.032	-12	14	-8
L. Caudate Nucleus (Head)		6.80	0.007	-9	2	7
R. Nucleus Accumbens/Caudate Nucleus (Head)		6.52	0.013	9	5	-5
L. Globus Pallidus		6.08	0.033	-12	2	-8
L. Midbrain		6.47	0.014	-6	-22	-5
L. Hypothalamus		5.95	0.042	6	-4	-11
L. + R. Thalamus		8.74	<0.001	0	-13	4
L. + R. Thalamus		8.43	<0.001	-6	-16	4
**Stop > Go**						
L. Cuneus	18	6.25	0.023	-12	-106	4
L. Inf. Occipital/Fusiform Gyrus	18/19	6.25	0.028	-39	-76	-14

The analysis included the entire pool of subjects. BA: Brodmann area; Inf: inferior; L: left hemisphere; Mid: middle; Post: posterior; R: right hemisphere; Sup: superior.

**Fig 3:**
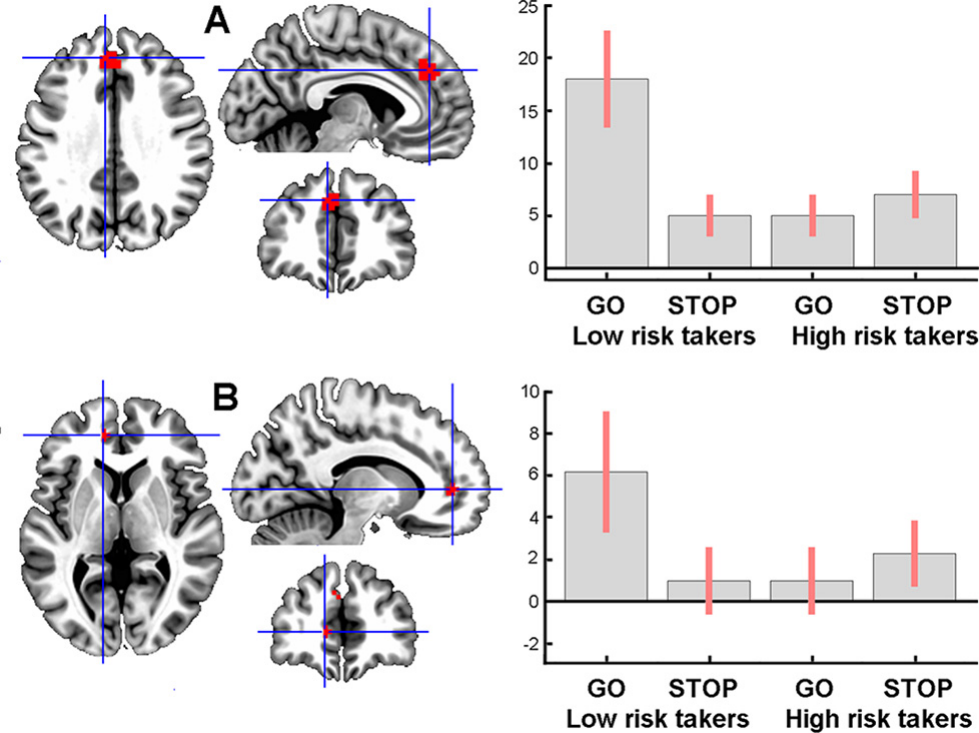
Areas of decision-related activation in BA 9 (A) and 10 (B) of the left MPFC in task performance-based groups. Corresponding plots show estimates of average effect size (ordinate, in an arbitrary unit) and SE within 6 mm spherical volumes centered at voxels with the highest values (indicated by crosshairs). The areas are shown overlaid onto three orthogonal slices of the MNI (ICBM152) template at the p < 0.0001 uncorrected threshold for better illustration.

The interaction between Group and Decision was not significant for questionnaire-based groups, or for grouping of subjects based on the score (1 and 2 vs. 3 and 4) of two ZKA-PQ items (#107 and 137, separately) with moderate D values for discrimination, while the interaction was significant for performance-based groups in two areas of the medial (M) PFC in the vicinity of the cingulate sulcus. One area had its peak (F_1, 25_ = 42.93, p = 0.029) at the MNI coordinates (x y z) -6 41 34 presumably corresponding to the left BA 9 (Fig 3A), and the other, more ventrally located area, peaking (F_1, 25_ = 40.37, p = 0.044) at the coordinates -12 47 4, appeared to correspond to the left medial part of BA 10 (Fig 3B). Post-hoc t-tests showed that the Go > Stop difference in activation was larger in the low than the high risk-taking group in both areas. Additional analyses performed within a ROI including two small volumes (see Materials and Methods) around the peaks revealed significant negative correlations between the Go > Stop activation difference and the number of Go decisions for both dorsal (t = 4.95, p = 0.001) and ventral (t = 5.76, p < 0.0001) MPFC areas for the entire sample. These effects, however, were not significant within groups.

The interaction between decision-related brain activation and the Peer factor was not significant in either questionnaire- or performance-based group, or in the entire pool of subjects combined across groups. The main effect of the Peer factor, as explored by t-tests using either Go > Baseline or Stop > Baseline contrasts, was also not significant for both Go and Stop conditions for either direction of comparison.

#### Outcome phase

**Fig 4:**
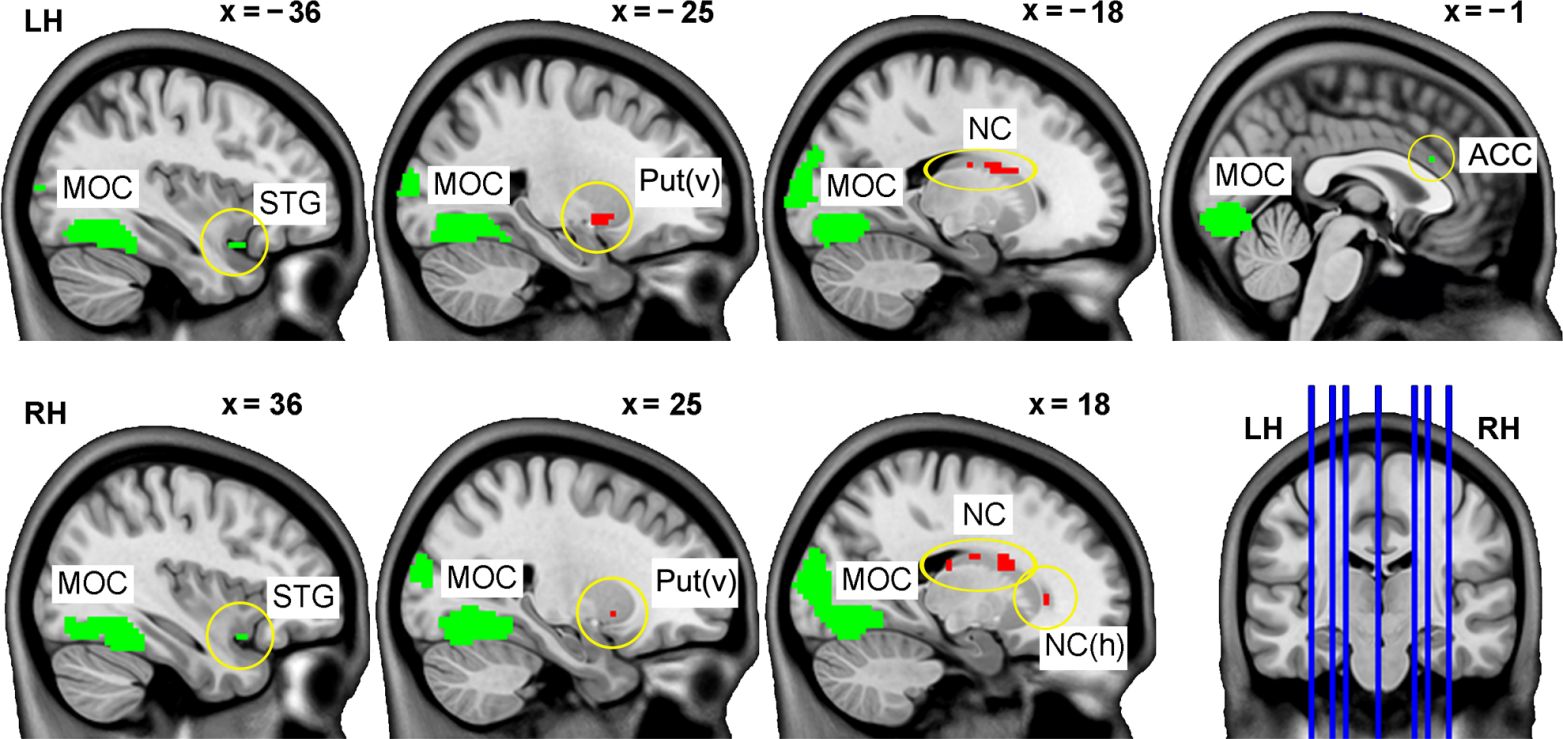
Areas of stronger (red) or weaker (green) activation for Pass than Crash outcome (p < 0.05, FWE corrected). The areas are shown overlaid onto sagittal slices of the MNI152 template specified with x coordinates above the slice images and in the lower right coronal image. Statistical and location details of the activation peaks are provided in Table 3. ACC: anterior cingulate cortex, LH: left hemisphere; MOC: medial occipital cortex; NC: body of caudate nucleus; Put(v): ventral putamen; RH: right hemisphere; STG: superior temporal gyrus.

**Fig 5:**
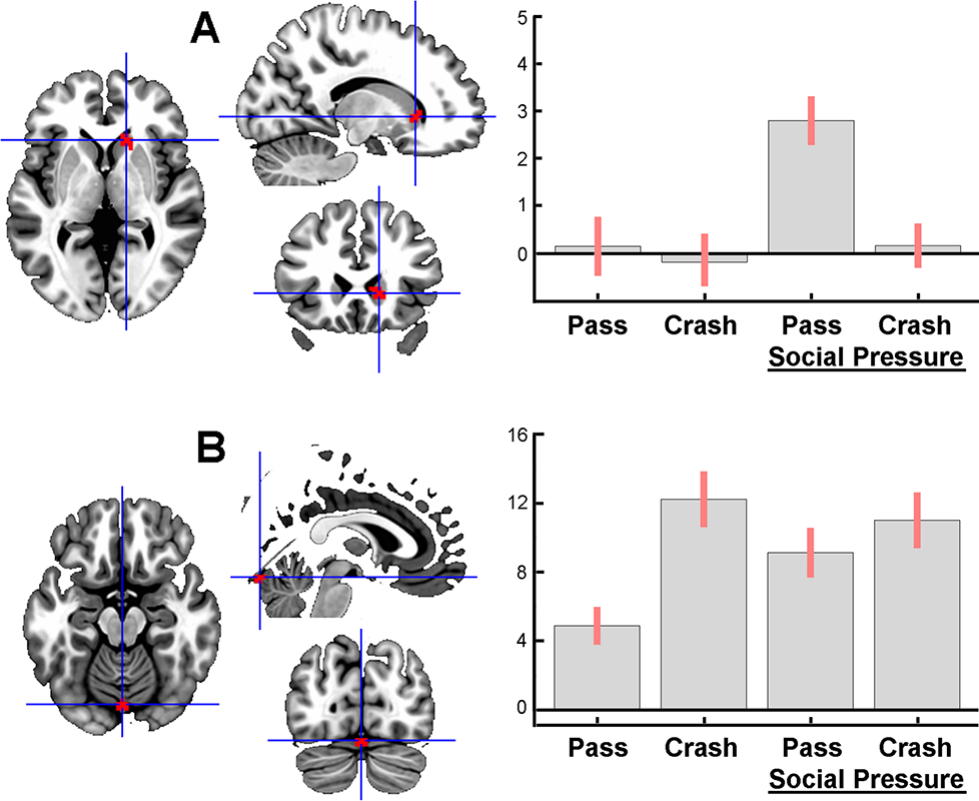
Areas of outcome-related activation manipulated by the social factor (peer competition) in the ventral part of the caudate head (A) and cerebellar vermis (B) obtained from the analysis of the entire sample (both high and low risk-takers together). Corresponding plots show estimates of average effect size (ordinate, in an arbitrary unit) and SE within 6 mm spherical volumes centered at voxels with the highest values (indicated by crosshairs). The areas are shown overlaid onto three orthogonal slices of the MNI (ICBM152) template at the p < 0.0001 uncorrected threshold for better illustration.

A comparison between Pass and Crash outcomes revealed activation areas mostly in the basal ganglia bilaterally including ventral putamen and in both dorsal and anterior ventral parts of the striatum (Fig 4, Table 3). Additionally, activation in the left lateral premotor and superior temporal cortical areas along with the left posterior thalamic and cerebellar activation was found. The opposite Crash > Pass contrast revealed large activation in the lateral and medial occipital areas in both hemispheres. Bilateral activation was also observed in the junction between temporal polar, inferior frontal, and insular cortices. Bilateral activations were also found in the inferior parietal cortex, right precuneus, and posterior middle temporal cortex. Non-significant trend (p = 0.057) was also observed in the opercular frontal-insular cortex. The interaction between Group and Decision was not significant for either questionnaire- or performance-based group. The interaction between outcome-related brain activation and the Peer factor was not significant in either questionnaire- or performance-based group. However, in the entire pool of subjects, areas in the caudate head (Fig 5A, coordinates 15 23 1; F_**1, 78**_ = 26.82, p = 0.035) and in the declive (vermis VI) of cerebellum (Fig 5B, coordinates 0–79–17; F_**1, 78**_ = 29.44, p = 0.015), showed significant, though different, interaction effects.

**Table 3:** Peak activation differences between conditions in the outcome phase.

t-contrast	BA	t	p _FWE-corr._	MNI coordinates		
Brain structure				x	y	z
**Pass > Crash**						
L. Precentral Gyrus	6	6.53	0.012	-66	-7	25
L. Superior Temporal Gyrus	22	7.61	0.001	-57	-13	4
R. Caudate, Head		6.42	0.016	18	29	4
R. Caudate, Head		6.02	0.036	12	26	-2
R. Nucleus Accumbens		6.10	0.031	21	5	-8
R. Caudate, Body		7.20	0.003	21	-22	22
R. Caudate, Body		8.14	<0.001	21	-7	25
R. Caudate, Body		7.80	0.001	18	8	25
R. Caudate, Body		6.92	0.006	21	14	19
L. Caudate, Body		6.89	0.006	-18	2	22
L. Caudate, Body		6.24	0.022	-18	-10	22
R. Putamen		6.07	0.032	24	8	-5
L. Putamen		6.67	0.009	-24	-1	-5
L. Thalamus, Pulvinar		6.33	0.019	-15	-31	16
L. Cerebellum (Crus I)		5.98	0.039	-39	-67	-35
**Crash > Pass**						
R. Ant. Cingulate Gyrus	24	6.12	0.029	3	23	25
R. Sup. Temporal Gyrus / Inf. Frontal Gyrus	38/47	6.48	0.014	36	14	-17
L. Sup. Temporal Gyrus	38/13	6.35	0.018	-36	11	-20
L. Inf. Frontal Gyrus / Insula*	47/13	5.79	0.057	-42	20	1
R. Mid. Temporal Gyrus	37	8.47	<0.001	-51	-58	4
R. Inf. Parietal Lobule	40	6.88	0.006	63	-40	25
L. Inf. Parietal Lobule	40	6.13	0.029	-66	-37	28
R. Precuneus	7	6.06	0.033	3	-52	67
R. Cuneus	19	5.88	0.048	9	-88	34
R. Cuneus	18/19	11.12	<0.001	21	-91	19
L. Cuneus	18	12.57	<0.001	-12	-103	4
R. Fusiform Gyrus	19	12.37	<0.001	30	-55	-14
R. Fusiform Gyrus	20/37	6.19	0.025	30	-31	-23
L. Fusiform Gyrus	19	12.10	<0.001	-30	-61	-14
L. Fusiform Gyrus	20	7.71	0.001	-30	-40	-23
R. Lingual Gyrus	18	10.50	<0.001	15	-76	-14
R. Lingual Gyrus	18	8.40	<0.001	27	-70	-8
R. Lingual Gyrus	18	8.15	<0.001	36	-73	-14
L./R. Lingual Gyrus	18	10.30	<0.001	0	-85	-8
L. Lingual Gyrus	17	10.45	<0.001	-6	-91	-11
L. Lingual Gyrus	18	9.69	<0.001	-30	-73	-11
L. Lingual Gyrus	18/19	9.46	<0.001	-21	-70	-11

The analysis included the entire pool of subjects.

*Non-significant trend.

## Discussion

### Questionnaire and behavioral data

We aimed to discriminate low and high risk-taking groups of male adolescents with personality tests, and compare their risk-taking behavior in a simulated driving task including a peer competition condition. The personality tests could not predict actual risk-taking behavior during task performance, whereas peer competition affected decision-making in both groups by having them take more risks and spend more time in making either Go or Stop decision. Performance-based groups showed a significant difference in Go response rates, suggesting that the grouping criterion could effectively divide the groups.

After Go responses (followed by Pass or Crash outcomes), Stop decision was made more often than Go decision, particularly after positive (Pass) outcomes, which was a rather unexpected result. It might suggest that subjects expected a higher probability of Crash after lucky Pass. The number of either decision was small after negative (Crash) outcomes, but this decrease in the number of either decision can be attributed to the small number of Crash trials (Table 1). Other interactions involving Group and Peer factors seem to reflect higher Go response rates in the high risk-taking group and the competition condition, combined with the small number of Crash trials.

The discrepancy between self-reported and performance-based measures of personality traits is not uncommon [29, 30], and can be particularly large in adolescents [31, 32]. In the present study, certain statements in the questionnaires might be rather difficult for teenage vocational school students to give accurate responses, as evidenced by those who asked the meaning of words during the test, such as oversensitive (ZKA-PQ #15, herkkänahkaisuuteen), impulsive (52, impulsiivisista), unconventional (147, epäsovinnaisesti), and rational (157, rationaalista). If the subjects’ general tendency for sensation-seeking was well reflected in the test scores, the Stoplight Game with monotonous scenery and vehicle progression (without acceleration) might not induce risk-taking consistently across subjects, making high scorers more easily get bored with the task and less motivated to take risks.

In addition, the ZKA-PQ items that represent sensation-seeking factor might not be optimal for predicting risky behavior. When two forms of impulsivity (sensation-seeking and acting without thinking), and their relations to risky behavior and executive function were examined [33], risky behavior was more strongly related to acting without thinking than sensation-seeking. Executive function was also negatively related to acting without thinking, while positively to sensation-seeking, suggesting that the rise in sensation-seeking during adolescence is not accompanied by a deficit in executive function.

It is often thought that risky behavior during adolescence implies weak executive function over behavior, and increased risky behavior in peer groups is due to insufficient prefrontal cognitive control compared with a more rapidly developing subcortical motivation system [17]. Evidence is yet sparse, but the positive correlation between sensation-seeking and working memory [33] or intelligence [34] suggests that those who exhibit stronger sensation-seeking drives are not less able to exert executive control over their behavior [35]. In further studies, it is worth assessing executive function or intelligence, and other forms of impulsivity to better understand their relations with adolescent risky behavior.

### MRI data

The study found differences in areas of activation between the decision types, and between the outcome types for entire pool of subjects. Furthermore, the effects of grouping on risky decision-related activation and the influence of peer competition on outcome-related activation were found.

We checked how brain activation at decision and outcome phases were modified by subjects’ propensity to take risks, as estimated with questionnaire or task performance. In line with the behavioral results, the fMRI analysis revealed significant interaction effects between group and decision type only in the groups formed by the task performance criterion. Neither way of grouping affected results for the outcome phase of the task. Thus, from here onward we consider only performance-based groups.

#### Decision phase

The direct Go > Stop comparison revealed a widespread pattern of activation, whereas the opposite Stop > Go contrast revealed only two occipital activation areas (Fig 2, Table 2). Indeed, Stop decision leads to a predictable consequence, while Go decision remains the situation uncertain with an equal probability of either outcome. The latter case, therefore, should be more demanding, also in terms of brain activation. Specifically, taking a risk presumes anticipation of a positive outcome that would justify the risk. Such anticipation was expectedly associated with the observed activation of the NAcc, a relay structure in the mesolimbic dopaminergic pathway. The NAcc has been considered as a key structure for reward anticipation [36], and its activation has been found to be proportional to the expected reward value [37, 38]. Co-activation of the NAcc (and neighboring caudate) with the ventral pallidum, thalamus, and midbrain is in agreement with known tight anatomical connections between these structures [39].

Two areas in the MPFC demonstrated activation only when risky decision was made by low but not by high risk-takers (Fig 3). In additional ROI analyses, a negative correlation between the Go > Stop activation level within these areas was observed for the entire sample, but not within groups, suggesting that it was a between-group rather than a group-nonspecific between-subject difference that contributed to the effect. This may suggest that Go decision-making involved a stronger conflict situation for low risk-takers, so that their higher level of MPFC activation reflected increased cognitive effort required to dare to take risks. While low risk-takers try harder to justify the risk, high risk-takers may have no hesitation that could be reflected by their faster response time. Previously, stronger MPFC activation (located similarly to the cluster in Fig 3B) has been found in low-risk gamblers in the High > Low risk contrast [16]. Although the anterior ACC has often been implicated in conflict-related allocation of cognitive resources [40], much evidence has been accumulated that the ACC may act in concert with neighboring parts of the MPFC [41, 42], as suggested by reciprocal anatomical connections between rostral parts of the ACC and medial BA 10 [43]. Moreover, the MPFC may have its own role in resolving conflicts or uncertainty. Thus, greater MPFC activation has been found when subjects were making a decision against their typical strategic bias [44], which reminds of the situation of low risk-takers making Go decisions in the present study. Because the dorsolateral (DL) PFC was implicated in maintenance of cognitive control in earlier studies [45], activation of this structure was expected in the decision phase of the task. Indeed, as revealed by both Go > Baseline and Stop > Baseline contrasts, the right DLPFC was activated during decision-making (see Supporting Information). However, we did not observe any difference in DLPFC activation depending on decision type or amount of risk-taking, or both factors.

#### Outcome phase

The Pass > Crash comparison revealed activation mostly in the basal ganglia (Fig 4). In particular, activation in the anterior and dorsal parts of the caudate nucleus was possibly related to excitation of action schemas based on the evaluation of action outcomes [46]. In our experiment, the outcome-related caudate activation was found exclusively in association with the rewarding (Pass) outcome.

Notably large medial occipital activation in the Crash > Pass contrast was due to a ‘realistic’ view of cracked windscreen appearing at the moment of crash, but not at successful passing. This visual inequality, assumingly, did not influence the activation related to cognitive aspects of the outcomes.

The Crash situation, as a negative outcome, expectedly activated ACC and parts of anterior inferior frontoinsular cortex. These structures appear to be jointly involved in internal generation of negative emotions, error detection, and initiation of adaptive responses to negative feedback [47–50]. Activation of the temporal polar cortex, bordering insular and inferior frontal cortices, could reflect importance of social/emotional meaning of the Crash event [51].

#### Peer influence

Although no group-specific effect of the social factor (peer competition) was found, analysis of the entire pool of subjects across groups revealed an interaction between social factor and outcome type. The effects were found in the head of the right caudate and in the cerebellar vermis (Fig 5). The activation in the caudate was specific to the combination of the Pass outcome and competition condition (Fig 5A). Indeed, the peer competition could increase a subjective value of the successful outcome. Therefore, the right caudate activation could reflect a satisfaction with a desired action in a socially loaded situation [52]. In fact, the location of this effect coincides with that observed in the Pass > Crash contrast. Thus, the head of the right caudate responded to the positive outcome of risky actions, and this response became even stronger when the social factor was added. Therefore, it appears that the caudate was involved both in integration of appetitive cues across the task phases and in modulation of this process by a social influence.

The other area where outcome-related activation was modulated by the social factor was found in the declive of cerebellar vermis. Its activation course across conditions (Fig 5B) can suggest that the competition condition made the positive outcome treated in the same way as the negative one. This could reflect changes in emotional value of the outcome. Indeed, the cerebellar vermis has often been referred to as a part of the ‘limbic cerebellum’, destruction of which affects emotions and personality both in primates and humans [53].

### Limitations

Finally, as limitations of the study, our sample is limited to male adolescents who are thought to be more prone to risk behavior than females, and the age ranges was limited to 18–19 years old by the ethics committee due to their relevance to driving behavior (e.g., driver’s license eligibility or driving experience). It should also be noted that our findings on risk-taking in a computerized driving game cannot be immediately generalized to real life.

We found that grouping of subjects by risk-taking propensities assessed with personality tests could not predict the differences in actual task performance or fMRI data. Previously, in the same subjects, we have found that only task performance-based, but not questionnaire-based, grouping led to between-group differences in fractional anisotropy of white matter [54]. Therefore, neither behavioral, nor functional or structural MRI data suggested any relevance between risk-taking propensities assessed by personality tests and actual risk-taking behavior of adolescents in the simulated driving task.

We assumed that the instructions to the subjects about the comparison of task performance results among peers would create sufficient social pressure comparable with actual presence of peers as in [17], and the effects were at least partly proven by more risky decisions and longer response time during peer competition. Furthermore, the social factor was always introduced in the last two sessions to make it a surprise, and thus time-related factors such as order effects could confound the data.

Given the fixed order and a short interval between Go decision and its outcomes (Pass or Crash), when convolved with a hemodynamic response function, corresponding regressors might share a substantial amount of variance leading to a problem of multicollinearity. It may particularly bias the results of subject-specific contrasts involving the Go decision. To avoid collinearity and improve distinction between Go decision and its outcome, the former was modelled as a condition with a variable duration according to response time (S4 Fig). We also created a jitter between yellow light onset and the moment of entering an intersection. Furthermore, we explicitly examined the multicollinearity issue by calculating bivariate coefficients of determination (R^2^) as the square of the correlation coefficient between the pair of regressors, convolved with the hemodynamic response function. Corresponding variance inflation factors (VIF = 1/(1-R^2^)) for session-wise pairs of relevant regressors in the model were also calculated. When regressors are orthogonal, VIF = 1, VIFs exceeding 10 are often considered harmful [55], while VIFs less than 5 are typically accepted as safe. The VIF values for both ‘Go and Pass’ (mean = 1.260, range: 1.028 ~ 2.247) and ‘Go and Crash’ (mean = 1.256, range: 1.007 ~ 2.514) pairs were below the recommended maximum level of 5.0, or even 2.5. In our case, only one session exceeded the level of 2.5 with the VIF value of 2.514 for the ‘Go and Crash’ pair. Therefore, multicollinearity appeared not to be an issue, suggesting that the parameter estimates were not substantially biased within sessions, making it even less problematic at the subject level and further at the second-level group analysis.

## Conclusions

There have been a number of studies on decision-making including risk-taking, but only very few have dealt with driving-related risk-taking in adolescents. Thus, in the present study, a driving situation was used in the form of a computer game, and the relationship between a risk-taking propensity and brain activation at the moment of risk-taking, as well as the influence of peer competition in male adolescents were of special interest. In addition, the same factors were analyzed for the outcome phase of the task.

A decision to take a risk activated the adolescent brain much more than a decision to stay safe. Confirming our behavioral results, not questionnaire-, but only task performance-based grouping was associated with between-group differences in activation. Thus, higher MPFC activation in low than in high risk-takers appeared to reflect a stronger conflict in making a risky decision, while the LPFC was equally involved in making either decision regardless of the propensity for risk-taking during task performance. The caudate nucleus expectedly demonstrated activation both when taking a risk and facing its positive outcome. Social pressure (peer competition) was associated with longer decision time, and further increased activation in the right caudate head but only for the rewarding outcome. This suggests an important role of the caudate in linking reward-directed risk-taking, reward processing, and social modulation of interaction of these two processes.

As we aimed at exploring risk-taking behavior and related brain activation, the findings on the relationship between grouping criteria and group differences in brain activation, as well as the roles of the MPFC and the caudate nucleus in mediating risk-taking and social aspects of risky driving behavior, respectively, can be considered as hallmarks of the present study.

## Supporting Information

S1 Text Analysis of activation commonalities between Decision types using Go > Baseline and Stop > Baseline contrasts. (DOCX)

S1 Fig Screen shots of task events at different phases of a single trial of the Stoplight game. A schematic track with intersections (horizontal lines) at varying distances is shown on the right bottom. Subjects made decisions after the traffic lights at the intersection turned yellow. Stop decision resulted in a short 3-s waiting with another car crossing from the side, while Go decision could result in either Pass with no delay or Crash with a longer 6-s delay. The game screen displayed time on the upper side counting down from 5 min. A progress bar on the left side showed the position of the car and the intersections on the track.
(TIF)

S2 Fig Distribution of low and high risk-taking groups according to questionnaire results. Circles mark 187 right-handed male respondents, and colored circles, either empty or filled, mark 43 high (red) and 46 low (blue) risk-takers asked to participate the experiment, and filled circles mark 17 subjects from each group. Dotted lines indicate the average scores of the tests (RPIS: mean = 29.73, SD = 4.049; ZKA-PQ: mean = 144.66, SD = 14.162).
(TIF)

S3 Fig Distribution of low and high risk-taking groups according to task performance. The positions of the circles are the same as in S2 Fig, and the subjects are now marked in the two groups according to the amount of risk-taking (Go response rate) during the task.
(TIF)

S4 Fig Plots of regressors (in an arbitrary unit) for Go (green) decision, and Pass (blue) and Crash (red) outcomes, convolved with a hemodynamic response function for a single fMRI session. The duration of the Go decision was modelled as response time [24] influencing the amplitude of blood flow responses and making the Go decision better distinguishable from the following Pass or Crash outcomes, and thus minimizing concerns about collinearity. There was also a jitter for the start of the Go decision but it was kept small (1.5–3.0 s) to prevent a possible dependency of decision time on interval between yellow light onset and the moment of entering an intersection.
(TIF)

S5 Fig Areas of activation (red) and deactivation (blue) common to both decision types. Activation areas were obtained as the overlap between the Go > Baseline and Stop > Baseline contrasts, and deactivation areas in the same way with the opposite contrasts. Before taking the overlap, the contrasts were thresholded at p < 0.05 voxel level after FWE correction. The areas are shown overlaid onto a series of sample axial slices of the MNI152 template specified with z coordinates above the slice image and in the lower right sagittal image. The right hemisphere corresponds to the right half of the axial images. Centers of mass for the clusters and the involved brain structures are detailed in Table A in S1 Text. ACC: anterior cingulate cortex; AIns: anterior insula; Crbl: cerebellum; DLPFC: dorsolateral prefrontal cortex; IOG: inferior occipital gyrus; IPL: inferior parietal lobule; MC: motor cortex; MOC: medial occipital cortex; MTG: middle temporal gyrus; PreC: precuneus; SMA: supplementary motor area; STG: superior temporal gyrus; STS: superior temporal sulcus; VLPFC: ventrolateral prefrontal cortex.
(TIF)

S6 Fig Areas of activation (red) and deactivation (blue) common to both outcome types. Activation areas were obtained as the overlap between the Pass > Baseline and Crash > Baseline contrasts, and deactivation areas in the same way with the opposite contrasts. Centers of mass for the clusters and the involved brain structures are detailed in Table B in S1 Text. Amg: amygdala; Isth: isthmus; MPFC: medial prefrontal cortex; PostCntG: postcentral gyrus; PCC: posterior cingulate cortex; PreMC: premotor cortex; SPL: superior parietal lobule; Th: thalamus.
(TIF)
