# Supplementary material for: Risk-Taking Behavior in a Computerized Driving Task: Brain Activation Correlates of Decision-Making, Outcome, and Peer Influence in Male Adolescents
Source: PLoS One. 2015 Jun 8;10(6):e0129516. doi: 10.1371/journal.pone.0129516 (PMC4460129; doi:10.1371/journal.pone.0129516)
Supplement: S1 Text — (DOCX) [file pone.0129516.s001.docx]

# Supporting Information

# Analysis of activation commonalities between Decision types using Go > Baseline and Stop > Baseline contrasts.

## Method

The areas of commonalities between conditions were extracted with the Marsbar toolbox (http://marsbar.sourceforge.net/) as the overlap between the decision types contrasted with Baseline, and between the outcome types contrasted with Baseline, both initially thresholded at p < 0.05 voxel level after family-wise error (FWE) correction.

## Results

### Decision phase

The comparison of Go and Stop decisions contrasted with Baseline revealed overlapping activation (S6 Fig., Table A) in the right dorsolateral (DLPFC) and ventrolateral (VLPFC) prefrontal cortices, bilateral anterior insula, bilateral posterior temporal cortex, and right inferior parietal cortex. Medial cortical activation was found in the right anterior cingulate, bilateral medial occipital cortex, and precuneus. Cerebellar activation was also found, more extended on the left side. Areas of deactivation were found in the lateral inferior occipital cortex bilaterally and in the left lateral and supplementary motor areas.

### Outcome phase

The comparison of Pass and Crash outcomes contrasted with Baseline showed overlapping activation (S7 Fig., Table B) in the bilateral anterior, posterior, and retrosplenial (isthmus) parts of the cingulate, and in the anterior medial prefrontal, posterior temporal, and medial occipital (cuneus) cortices. Bilateral activation was also found in the amygdala and cerebellum. Only left-sided activation was found in the VLPFC and intraparietal sulcus. Outcome-related deactivation was found in the right precuneus and bilaterally in the anterior insular, postcentral, premotor cortices including pre-supplementary motor area, and medial thalamus.

## Discussion

### Decision phase

The decision phase of the experiment expectedly involved the LPFC, among other structures (S6 Fig.). Thus, making either Go or Stop decision was associated with activation in the right DLPFC (BA 9, 10, 46), right VLPFC (BA 45, 47), and bilateral anterior insula (BA 13). As has been revealed by a meta-analysis of studies using Go/No-Go and Stop Signal paradigms [S1], the right DLPFC, VLPFC, and anterior insula activation were most specific to motor response inhibition, a key cognitive control function. Thus, activation of the same set of structures in our study could reflect a competition between alternative responses by inhibiting one in favor of the other. These results also suggest that involvement of the right LPFC is equally needed no matter what type of decision is made.

### Outcome phase

In our experiment, both positive (Pass) and negative (Crash) outcomes were potentially emotion-triggering events, and thus the finding of bilateral amygdalar activation for either outcome was not surprising (S7 Fig.). Although the amygdala is most known to mediate negative, aversive emotions, it has been found that different sub-nuclei within the amygdalar complex respond to either aversive or appetitive stimuli, and amygdalar activation related to positive emotions has been documented as well [S2, S3]. Co-activation of amygdalae with bilateral parahippocampal, middle temporal cortex, and left LPFC could indicate formation of emotional memory [S4]. In addition, parts of the ACC and medial (M) PFC were also activated by either outcome. Similarly located MPFC activation areas along with activation in the left amygdala have been found to encode a reward value in a gambling task [41].

## References

S1. Levy BJ, Wagner AD. Cognitive control and right ventrolateral prefrontal cortex: reflexive reorienting, motor inhibition, and action updating. Ann N Y Acad Sci. 2011; 1224: 40-62.

S2. Ernst M, Pine DS, Hardin M. Triadic model of the neurobiology of motivated behavior in adolescence. Psychol Med. 2006; 36: 299-312.

S3. Hamann SB, Ely TD, Hoffman JM, Kilts CD. Ecstasy and agony: activation of the human amygdala in positive and negative emotion. Psychol Sci. 2002; 13: 135-141.

S4. Murty VP, Ritchey M, Adcock RA, LaBar KS. fMRI studies of successful emotional memory encoding: a quantitative meta-analysis. Neuropsychologia. 2010; 48: 3459-3469.

**Table A. Areas of activation changes in the decision phase irrespective of decision types.** The areas were calculated as the overlap of two t-statistic maps, both thresholded at p < 0.05 voxel level after FWE correction. The coordinates represent centers of mass of the clusters and that some clusters spread beyond a single brain structure. BA: Brodmann area; Inf: inferior; L: left hemisphere; Mid: middle; Post: posterior; R: right hemisphere; Sup: superior

| **Overlapping contrasts** | BA | MNI coordinates | | |
| --- | --- | --- | --- | --- |
| Brain structure |  | x | y | z |
| **Go > Baseline and Stop > Baseline** |  |  |  |  |
| L. Insula | 13 | -38 | 13 | -6 |
| R. Inf. Frontal Gyrus | 45 |  |  |  |
| + Mid. Frontal Gyrus | 46 | 45 | 20 | 16 |
| R. Insula + Inf. Frontal Gyrus | 13, 47 | 45 | 26 | -1 |
| R. Mid. Frontal Gyrus | 10 | 27 | 60 | 26 |
| R. Mid. Frontal Gyrus | 9 | 38 | 16 | 40 |
| R. Mid. Frontal Gyrus | 9 | 36 | 13 | 37 |
| R. Ant. Cingulate | 32 | 7 | 43 | 11 |
| R. Inf. Parietal Lobule | 40 | 48 | -49 | 40 |
| R. Inf. Parietal Lobule | 40 | 50 | -40 | 44 |
| R. Inf. Parietal Lobule | 40 | 35 | -58 | 47 |
| R. Inf. Parietal Lobule | 40 | 57 | -33 | 42 |
| R. Precuneus | 7 | 3 | -47 | 46 |
| R. Postcentral Gyrus | 3 | 18 | -40 | 76 |
| L. Postcentral Gyrus | 3 | 23 | -39 | 73 |
| L. Sup. Temporal Gyrus | 22 | -61 | -45 | 16 |
| R. Mid. Temporal Gyrus | 21 | 54 | -26 | -7 |
| R.+L. Cuneus, Lingual Gyrus, | 17, 18, 19 |  |  |  |
| and L. Cerebellum (Lobule V, Crus I) |  | 2 | -68 | 4 |
| R. Cerebellum (lobule VI) |  | 37 | -52 | -29 |
| **Baseline > Go and Baseline > Stop** |  |  |  |  |
| L. SMA / Pre-SMA | 6 | -7 | -1 | 55 |
| L. Central Sulcus | 4 | -35 | -25 | 57 |
| R. Inf. Occipital Gyrus | 18 | 28 | -98 | -6 |
| R. Inf. Occipital Gyrus | 18/19 | 37 | -90 | -7 |
| L. Inf. Occipital Gyrus | 18 | -32 | -96 | -7 |

**Table B. Areas of activation changes in the outcome phase irrespective of outcome type.**

| **Overlapping contrasts** | BA | MNI coordinates | | |
| --- | --- | --- | --- | --- |
| Brain structure |  | x | y | z |
| **Pass > Baseline** and **Crash > Baseline** |  |  |  |  |
| L. + R. Med. Frontal Gyrus + Ant. Cingulate | 9, 10, 32 | 1 | 56 | 18 |
| R. Ant. Cingulate | 33/24 | 7 | 33 | 5 |
| L. Inf. Frontal Gyrus | 45/47 | -51 | 29 | 6 |
| L. Sup. Temporal Sulcus | 39 | -53 | -62 | 26 |
| L. Sup. Temporal Sulcus | 21 | -51 | -26 | -4 |
| R. Mid. Temporal Gyrus | 21 | 59 | -40 | -1 |
| L. Mid. Temporal Gyrus | 21 | -60 | -45 | -1 |
| L. Inf. Temporal Gyrus | 37/20 | -51 | -54 | -16 |
| L. Intraparietal Sulcus | 7/40 | -36 | -66 | 44 |
| R. Amygdala |  | 29 | -1 | -18 |
| L. Amygdala |  | -26 | -4 | -22 |
| R. Lingual Gyrus + L. Precuneus |  |  |  |  |
| + L./R. Posterior Cingulate | 18, 31 | 4 | -86 | -17 |
| L./R. Cuneus | 18 | 0 | -81 | 24 |
| R. Cerebellum (Crus I) |  | 46 | -58 | -28 |
| L. Cerebellum (Lobule VI) |  | -30 | -44 | -28 |
| R. Cerebellum (Lobule VI) |  | 19 | -59 | -18 |
| L. Cerebellum (Lobules V, VI + Crus I) |  |  |  |  |
| + L. Isthmus | 30 | -20 | -65 | -20 |
| R. Isthmus | 30 | 9 | -46 | 0 |
| **Baseline > Pass** and **Baseline > Crash** |  |  |  |  |
| L. Sup. Frontal Gyrus | 6 | -18 | -4 | 70 |
| R. Sup. Frontal Gyrus | 6 | 18 | 0 | 66 |
| L. Precentral Sulcus | 6 | -35 | -7 | 53 |
| L. + R. Pre-SMA | 6 | 0 | 7 | 53 |
| L. Cingulate Sulcus | 24/31 | -11 | -21 | 44 |
| L. Ant. Insula | 13 | -29 | 24 | 5 |
| R. Ant. Insula | 13 | 33 | 25 | 4 |
| L. Marginal Sulcus | 5/7 | -13 | -45 | 56 |
| L. Sup. Parietal Lobule | 7 | -15 | -56 | 64 |
| R. Sup. Parietal Lobule | 7 | 14 | -55 | 61 |
| L. Precuneus | 7 | -15 | -74 | 43 |
| R. Precuneus | 7 | 20 | -73 | 42 |
| L. Thalamus |  | -6 | -18 | 0 |
| R. Thalamus |  | 6 | -14 | 0 |
