# Supplementary figures and images for: Risk-Taking Behavior in a Computerized Driving Task: Brain Activation Correlates of Decision-Making, Outcome, and Peer Influence in Male Adolescents
Source: PLoS One. 2015 Jun 8;10(6):e0129516. doi: 10.1371/journal.pone.0129516 (PMC4460129; doi:10.1371/journal.pone.0129516)

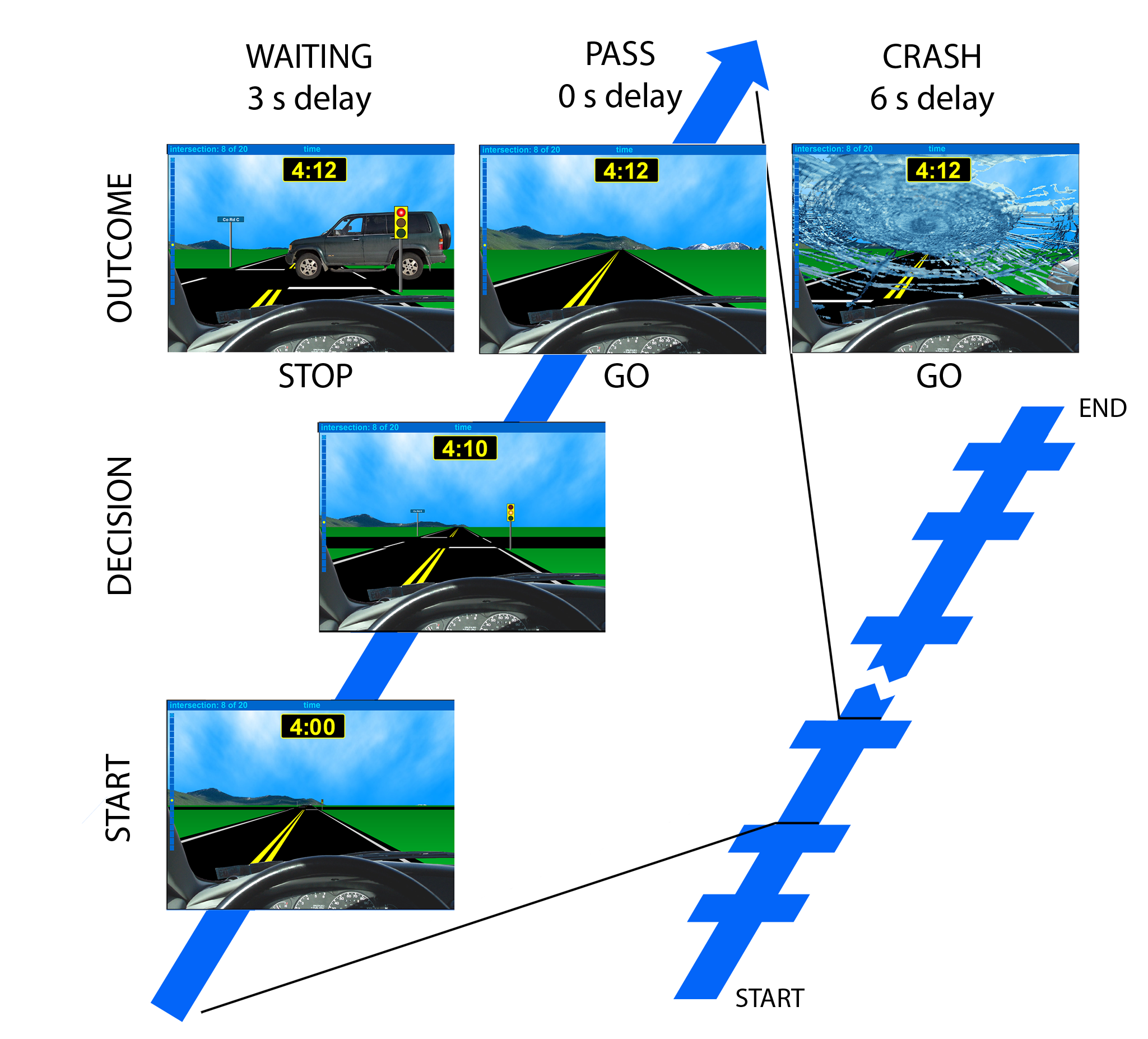

Supplement: S1 Fig — A schematic track with intersections (horizontal lines) at varying distances is shown on the right bottom. Subjects made decisions after the traffic lights at the intersection turned yellow. Stop decision resulted in a short 3-s waiting with another car crossing from the side, while Go decision could result in either Pass with no delay or Crash with a longer 6-s delay. The game screen displayed time on the upper side counting down from 5 min. A progress bar on the left side showed the position of the car and the intersections on the track. (TIF) [file pone.0129516.s002.tif]

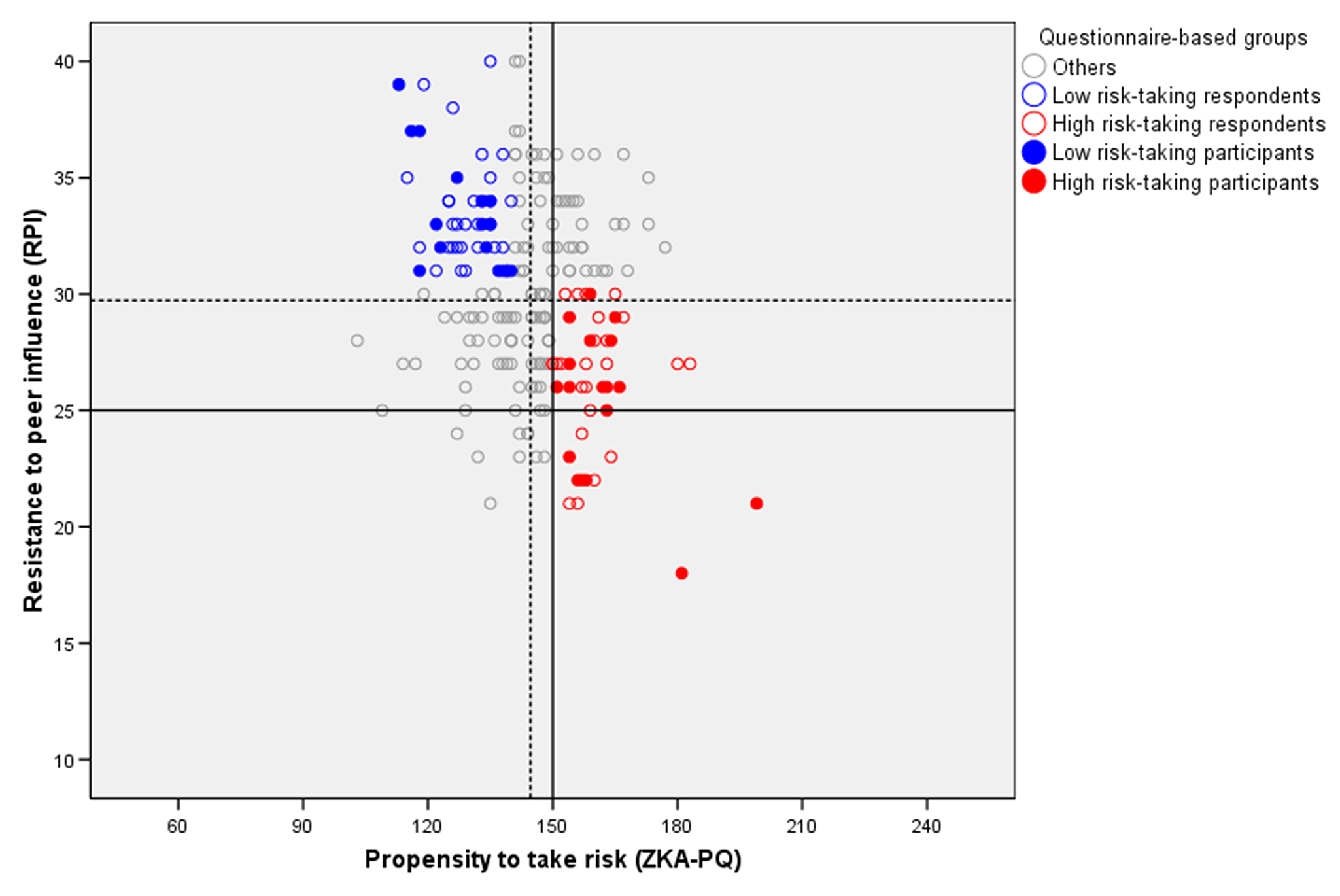

Supplement: S2 Fig — Circles mark 187 right-handed male respondents, and colored circles, either empty or filled, mark 43 high (red) and 46 low (blue) risk-takers asked to participate the experiment, and filled circles mark 17 subjects from each group. Dotted lines indicate the average scores of the tests (RPIS: mean = 29.73, SD = 4.049; ZKA-PQ: mean = 144.66, SD = 14.162). (TIF) [file pone.0129516.s003.tif]

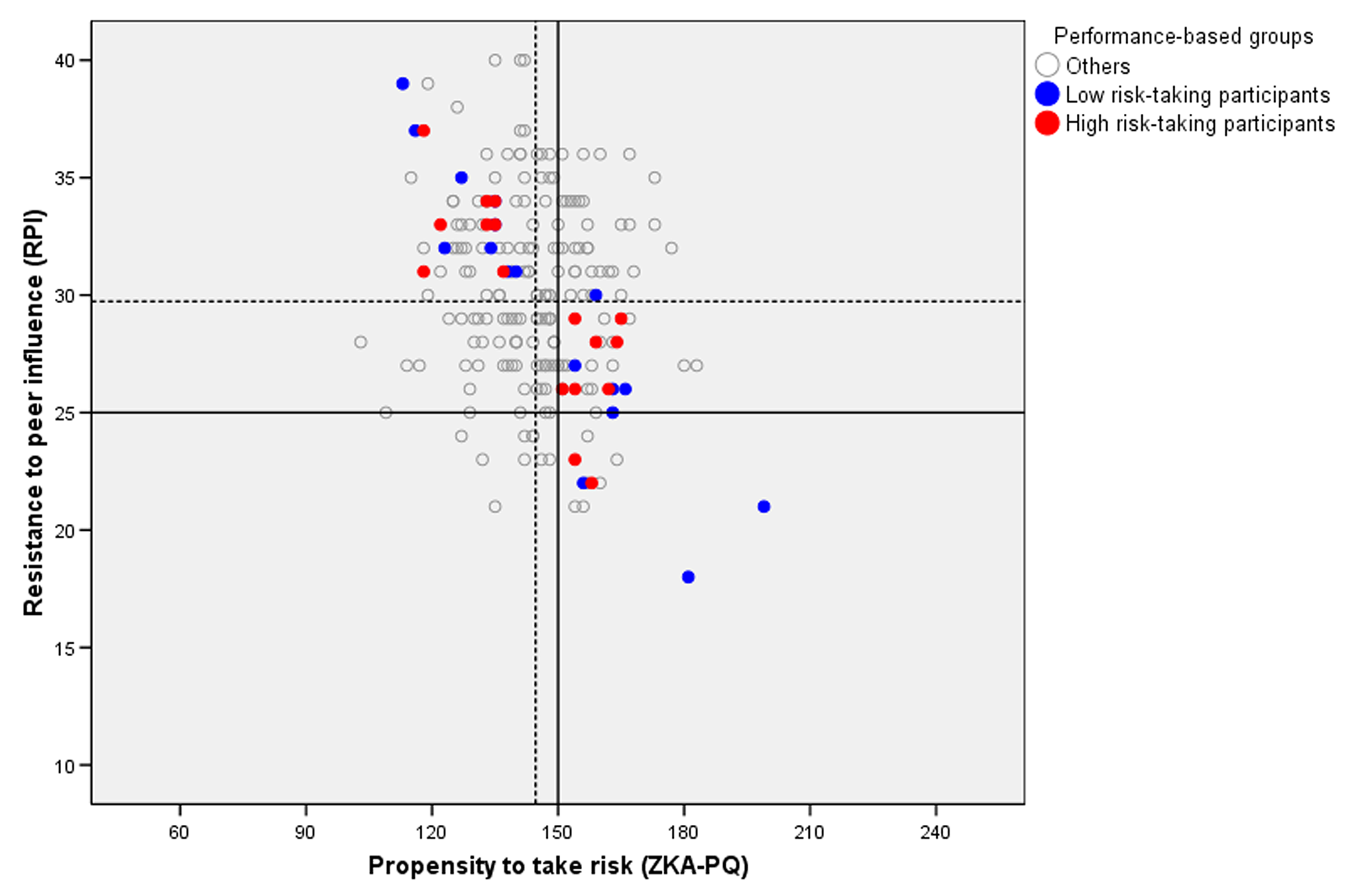

Supplement: S3 Fig — The positions of the circles are the same as in S2 Fig, and the subjects are now marked in the two groups according to the amount of risk-taking (Go response rate) during the task. (TIF) [file pone.0129516.s004.tif]

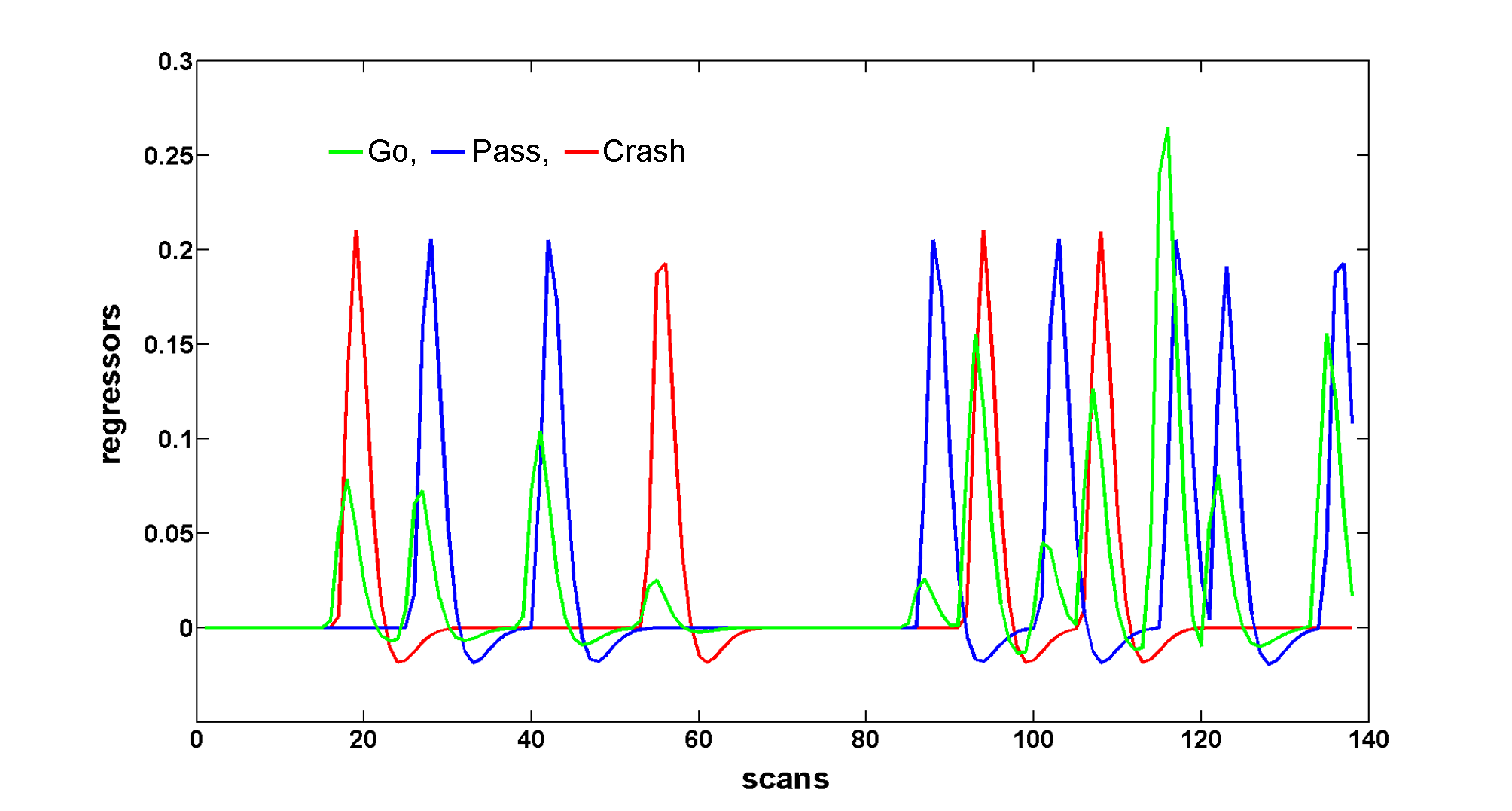

Supplement: S4 Fig — The duration of the Go decision was modelled as response time [24] influencing the amplitude of blood flow responses and making the Go decision better distinguishable from the following Pass or Crash outcomes, and thus minimizing concerns about collinearity. There was also a jitter for the start of the Go decision but it was kept small (1.5–3.0 s) to prevent a possible dependency of decision time on interval between yellow light onset and the moment of entering an intersection. (TIF) [file pone.0129516.s005.tif]

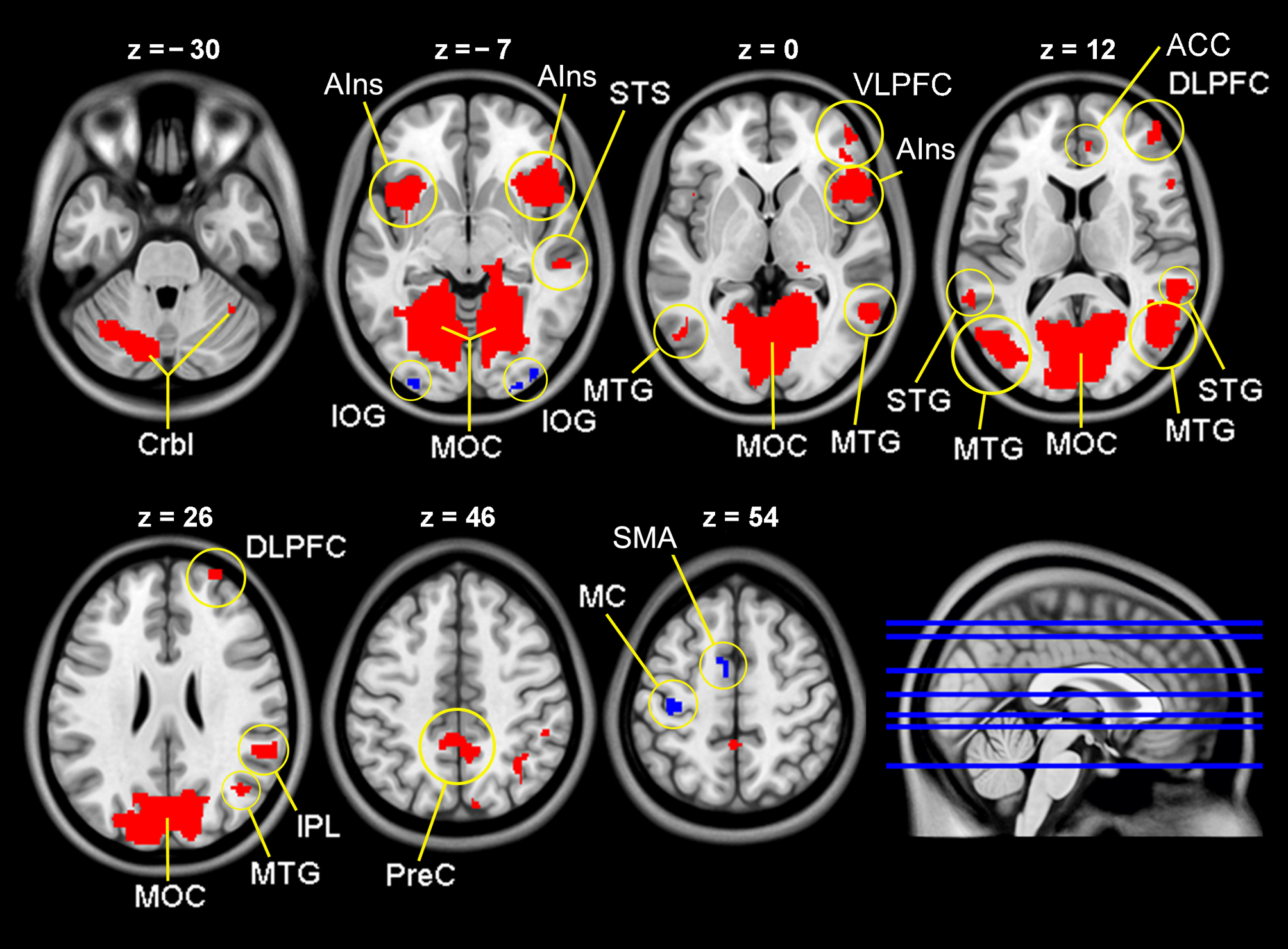

Supplement: S5 Fig — Activation areas were obtained as the overlap between the Go > Baseline and Stop > Baseline contrasts, and deactivation areas in the same way with the opposite contrasts. Before taking the overlap, the contrasts were thresholded at p < 0.05 voxel level after FWE correction. The areas are shown overlaid onto a series of sample axial slices of the MNI152 template specified with z coordinates above the slice image and in the lower right sagittal image. The right hemisphere corresponds to the right half of the axial images. Centers of mass for the clusters and the involved brain structures are detailed in Table A in S1 Text. ACC: anterior cingulate cortex; AIns: anterior insula; Crbl: cerebellum; DLPFC: dorsolateral prefrontal cortex; IOG: inferior occipital gyrus; IPL: inferior parietal lobule; MC: motor cortex; MOC: medial occipital cortex; MTG: middle temporal gyrus; PreC: precuneus; SMA: supplementary motor area; STG: superior temporal gyrus; STS: superior temporal sulcus; VLPFC: ventrolateral prefrontal cortex. (TIF) [file pone.0129516.s006.tif]

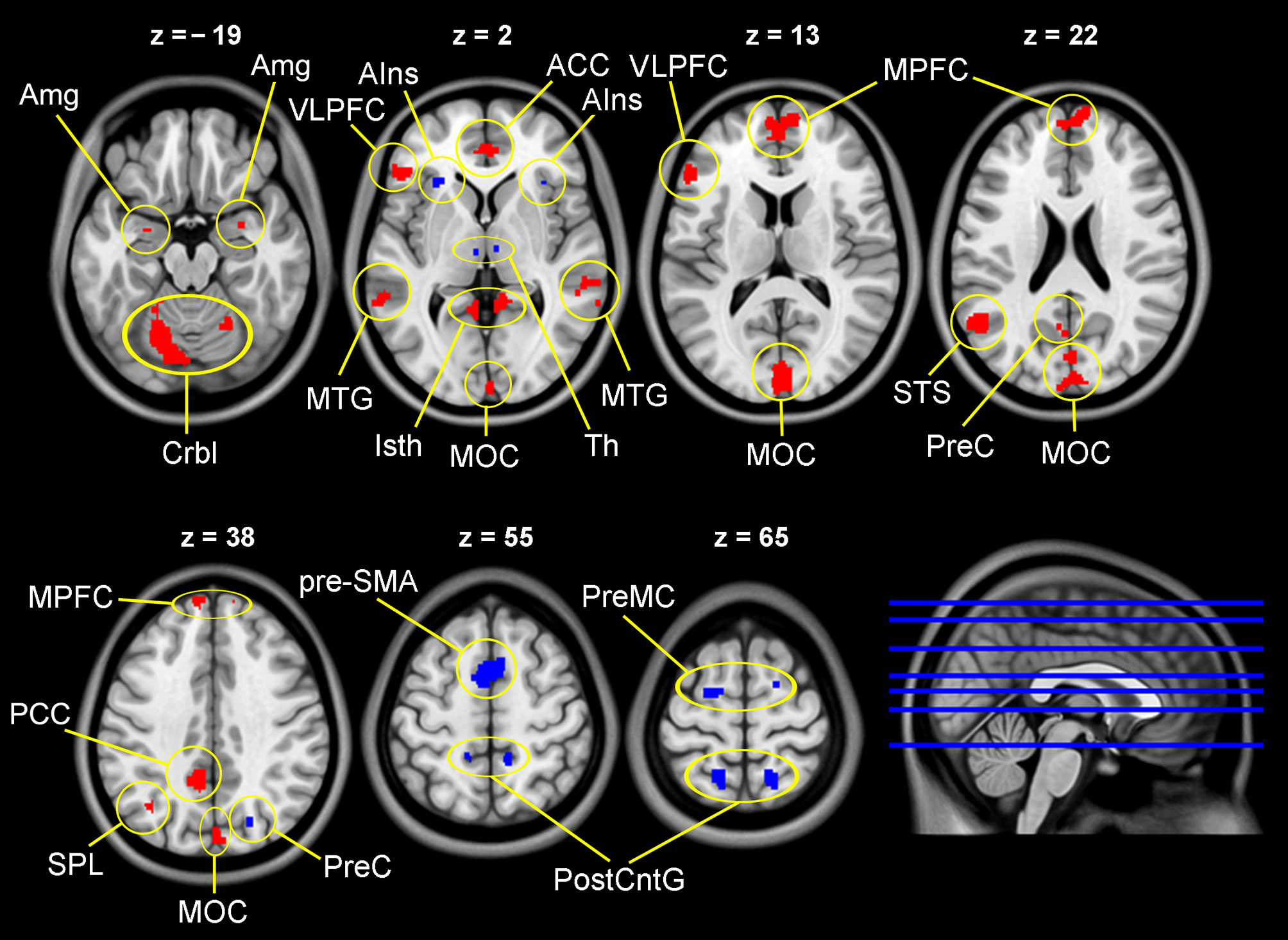

Supplement: S6 Fig — Activation areas were obtained as the overlap between the Pass > Baseline and Crash > Baseline contrasts, and deactivation areas in the same way with the opposite contrasts. Centers of mass for the clusters and the involved brain structures are detailed in Table B in S1 Text. Amg: amygdala; Isth: isthmus; MPFC: medial prefrontal cortex; PostCntG: postcentral gyrus; PCC: posterior cingulate cortex; PreMC: premotor cortex; SPL: superior parietal lobule; Th: thalamus. (TIF) [file pone.0129516.s007.tif]
